# Influence of Temperature on Mechanical Properties of P(BAMO-r-THF) Elastomer

**DOI:** 10.3390/polym12112507

**Published:** 2020-10-28

**Authors:** Jinxian Zhai, Hanpeng Zhao, Xiaoyan Guo, Xiaodong Li, Tinglu Song

**Affiliations:** 1 School of Materials, Beijing Institute of Technology, Beijing 100081, China; gxy@bit.edu.cn (X.G.); bitlxd@bit.edu.cn (X.L.); 2 China Academy of Railway Sciences, Standards and Metrology Research Institute, Beijing 100081, China; crcczhaohp@126.com; 3 Experimental center of advanced materials, School of Materials, Beijing Institute of Technology, Beijing 100081, China; song@bit.edu.cn

**Keywords:** P(BAMO-r-THF) elastomer, BAMO micro-block, aggregation morphology, mechanical properties, strain-induced crystallization

## Abstract

The relationship between temperature and the mechanical properties of an end cross-linked equal molar random copolyether elastomer of 3,3-bis(azidomethyl)oxetane and tetrahydrofuran (P(BAMO-r-THF)) was investigated. During this investigation, the performances of two P(BAMO-r-THF) elastomers with different thermal histories were compared at different temperatures. The elastomer as prepared at 20 °C (denoted as S0) exhibited semi-crystallization morphology. Wide angle X-ray diffraction analysis indicated that the crystal grains within elastomer S0 result from the crystallization of BAMO micro-blocks embedded in P(BAMO-r-THF) polymeric chains, and the crystallinity is temperature irreversible under static conditions. After undergoing a heating-cooling cycle, this elastomer became an amorphous elastomer (denoted as S1). Regarding mechanical properties, at 20 °C, break strains and stresses of 315 ± 22% and 0.46 ± 0.01 MPa were obtained for elastomer S0; corresponding values of 294 ± 6% and 0.32 ± 0.02 MPa were obtained for elastomer S1. At −40 °C, these strains and stresses simultaneously increased to 1085 ± 21% and 8.90 ± 0.72 MPa (S0) and 1181 ± 25% and 10.23 ± 0.44 MPa (S1), respectively, owing to the strain-induced crystallization of BAMO micro-blocks within the P(BAMO-r-THF) polymeric chains.

## 1. Introduction

Solid rocket propellants are a type of composites composed of (among others) polymeric binders, curing agents, high-energy additives, oxidizers, metallic fuel additives, and burning rate modifiers. The binders are generally used to bind together other ingredients to form a tough elastomeric three-dimensional structure [1]. Polymeric binders with excellent mechanical properties can effectively absorb, dissipate exterior applied load, and maintain propellant structural integrity under various intricate conditions. Compared with traditional hydroxyl-terminated polybutadiene inert binders, energetic binders can enhance the energy level as well as increase the burning rate and specific impulse of propellant systems. That is, these binders release additional heat and lead to an increase in the temperature of pyrolysis and combustion, and hence, have attracted considerable attention [2].

Energetic azido polymeric binders, such as glycidyl azido polymer (GAP), poly[(3-azidomethyl)methyloxetane] (PAMMO), and poly(3,3-bis(azidomethyl)oxetane) (PBAMO), have been extensively investigated because azido bond decomposition is associated with the evolution of a large amount of energy (355 kJ mol^−1^) [3,4,5]. In fact, PBAMO is quite attractive, owing to the high energy arising from the two –N_3_ bonds in the monomer structure. Unfortunately, due to the symmetry of the BAMO monomer and the stereo-regularity of the BAMO homo-polymeric chain, PBAMO is prone to crystallization, exhibits a relatively high glass transition temperature (T_g_), and is solid at room temperature. This results in inadequate mechanical properties (break strain: ~10%), thereby severely limiting the practicality of PBAMO [6,7,8].

Randomly introducing a second monomer into a homo-polymerization chain structure, constructing irregular segment structures, and increasing the configuration entropy represent an effective approach of inhibiting polymer crystallinity [9]. The copolymerization of BAMO with other cyclic ether monomers by means of cationic ring opening polymerization is a simple and effective method of improving the practicality of PBAMO. Petrov et al. found that the crystallinity of BAMO/AMMO random copolyethers (P(BAMO-r-AMMO)) decreases gradually with increasing AMMO content. When AMMO and BAMO monomers are equimolar and are homogeneously distributed within P(BAMO-r-AMMO) polymeric chains, the crystallinity of BAMO micro-blocks can be effectively restrained [10]. However, the bulky side group of AMMO monomer is disadvantageous to decreasing the polymer glass transition temperature [11], and hence, the T_g_ of the resulting P(BAMO-r-AMMO) is approximately −35 °C [12]. Similarly, owing to the bulky side groups of 3-nitratomethyl-3-methyloxetane (NMMO), a BAMO/NMMO random copolymer is characterized by a T_g_ of −23 °C. These are unfavorable to the mechanical properties, especially at low temperature [13].

**Scheme 1:**
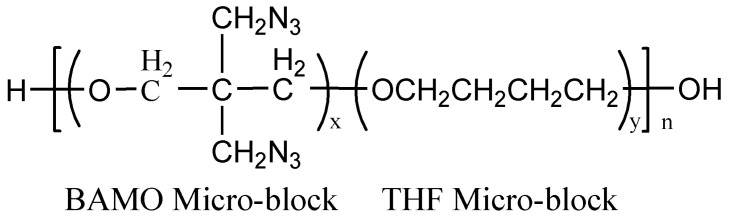
Molecular structure of prepolymer P(BAMO-r-THF).

Poly(tetrahydrofuran) (PTHF) is composed of a flexible chain structure and is often used to tailor the low-temperature mechanical properties of crystalline polymers [14,15]. The equal molar random copolyether of BAMO with THF (P(BAMO-r-THF), see Scheme 1) exists as a liquid at room temperature and is characterized by a T_g_ of approximately −60 °C, simultaneously meeting energy and mechanical-property requirements [16]. Correspondingly, some applied studies, such as P(BAMO-r-THF) propellant thermal decomposition, combustion, and performance modification, have been intensively reported [17,18,19]. However, to date, few studies on the mechanical behaviors of P(BAMO-r-THF) binders have been reported.

Exploring the mechanical behaviors of P(BAMO-r-THF) binders is essential to the use of these binders in practical applications. In a propellant system, a P(BAMO-r-THF) binder is end-reacted with a multifunctional curing agent, forming a three-dimensional crosslinking network and bearing applied loads [1]. In the present study, a hydroxyl terminated equal molar random copolyether of P(BAMO-r-THF) and polyisocyanate N100 (hexamethylene diisocyanate-H_2_O adduct) were used as a binder and a cross-linker, respectively [20]. These components were used to prepare urethane end-crosslinked P(BAMO-r-THF) elastomers based on the reaction between terminal hydroxyl and isocyanate. The relationship between temperature and the mechanical behaviors of the elastomers was systematically investigated.

## 2. Experimental

### 2.1. Materials

A hydroxyl terminated equal molar random copolyether of 3,3-bis(azidomethyl) oxetane with tetrahydrofuran (P(BAMO-r-THF), hydroxyl value: 0.36 mmol g^−1^, number average molecular weight: 5600 g mol^−1^, polydispersity index: 1.72) and N100 (isocyanate concentration: 5.25 mmol g^−1^, functionality: 3.9), were provided by Liming Research Institute of Chemical Industry (Luoyang, China) and used as an energetic binder and a polyisocyanate cross-linker, respectively. Furthermore, dibutyltin dilaurate (Alfa Aesar, Tianjin, China) were purchased and used as a curing catalyst.

### 2.2. Elastomer Preparation

Utilizing an equivalent molar ratio of isocyanate comprising N100 to hydroxyl comprising prepolymer P(BAMO-r-THF), all components were uniformly mixed according to the composition listed in Table 1, and degassed under vacuum. The mixture was cured at 20 °C until the isocyanate groups disappeared completely, as indicated by Fourier transform infrared spectroscopy. The resultant elastomer was kept at 20 °C and was denoted as S0. To eliminate the kinetic influence of changes in elastomer aggregation, the partial elastomer (S0) was heated to 50 °C and held for 30 min. Afterward, the elastomer was held at 20 °C, and the resulting elastomer was denoted as S1.

### 2.3. Characterization

Non-isothermal differential scanning calorimetry (DSC) was used to characterize the temperature thermal-response of end-crosslinked P(BAMO-r-THF) elastomers on an F204 instrument (Netzsch, Bavaria, Germany) equipped with a refrigerated cooling system. Temperature calibration was performed using an indium standard (T_m_ = 156.6 °C). Using liquid nitrogen, elastomer samples weighing 10 mg were quenched to from 20 °C to −100 °C. Afterward, DSC tests were performed at a heating rate of 2 K min^−1^ under a nitrogen atmosphere.

The morphology of P(BAMO-r-THF) elastomers was observed via polarizing optical microscopy (POM; Olympus BX51, Olympus Corporation, Tokyo, Japan). The microscope was equipped with a computer-controlled CCD camera and a thermal platform (THMS600, Linkam, Tadworth, UK). The P(BAMO-r-THF) elastomer films (thickness: 50 µm) were sandwiched between two clean glass slides and then quenched from 20 °C to −50 °C. The specimens were kept at the designated temperatures for 30 min, and then POM micrographs were captured at temperatures ranging from low to high.

Wide-angle X-ray diffraction (WAXD) measurements of P(BAMO-r-THF) elastomers were conducted on an automatic powder diffractometer (D8 Advance, Bruker-AXS, Karlsruhe, Germany; Ni-filtered Cu Kα radiation (40 kV, 40 mA)) equipped with a thermal platform. WAXD profiles were obtained in a 2θ scan mode for 2θ ranging from 5° to 40°. Prior to these measurements, the elastomer samples were quenched from 20 to −50 °C. From low to high temperature, the specimens (dimensions: 13 × 8 × 1 mm^3^) were held for 30 min at the selected temperature, and the WAXD measurements were performed at a scanning rate of 5° min^−1^.

We obtained load-extension curves of P(BAMO-r-THF) elastomers through uniaxial stretching on a mechanical tester (Instron 6022, Instron Corporation, Eden Prairie, MN, USA). Elastomer specimens were cut to a dumbbell shape (dimensions: 100 × 20 × 5 mm^3^). Subsequently, the specimens were fixed in the mechanical tester, and kept at the designated drawing temperature for 30 min, allowing temperature equilibration prior to the drawing process. The mechanical tests were conducted at a constant cross-head strain rate of 20 mm·min^−1^.

## 3. Results and Discussion

### 3.1. Thermal Properties

**Figure 1:**
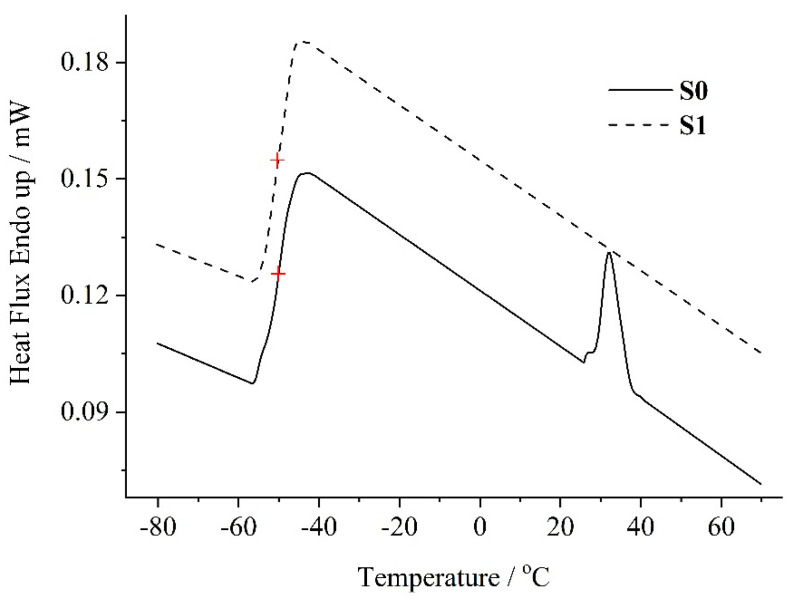
Differential scanning calorimetry (DSC) curves of P(BAMO-r-THF) elastomers.

The non-isothermal differential scanning calorimetric (DSC) curves of P(BAMO-r-THF) elastomers S0 and S1 are shown in Figure 1. In each case, a step peak occurs at approximately −50 °C, corresponding to the glass transition temperature of each elastomer [21]. For temperatures ranging from 25 to 38 °C, elastomer S0 gives rise to a sharp endothermic peak, whereas elastomer S1 gives rise to a straight line, without a perceptible thermal effect. This indicates that after elastomer S0 undergoes a heating-cooling treatment, the resulting elastomer (i.e., S1) exhibits completely different thermal behavior.

### 3.2. Aggregation Morphologies

**Figure 2:**
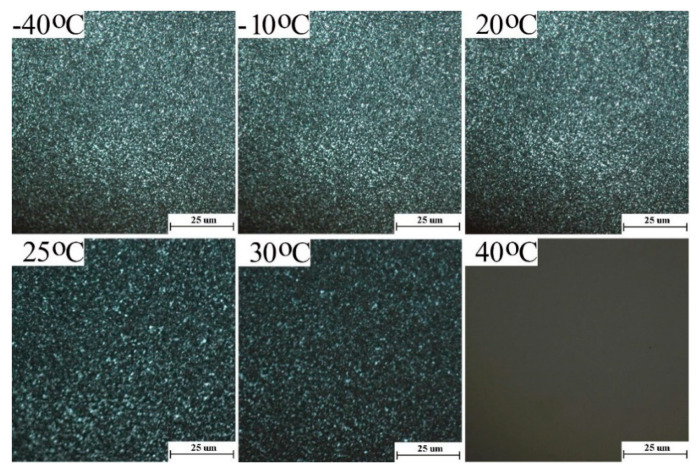
Polarizing optical microscopy (POM) snapshots of elastomer S0 at different temperatures.

Through polarizing optical microscopy (POM), the aggregation morphologies associated with different thermal histories of P(BAMO-r-THF) elastomers S0 and S1 are continuously observed for temperatures ranging from low to high. The POM snapshots of elastomer S0 at different temperatures (see Figure 2) reveal that, at temperatures below 40 °C, many small bright patches occur in the elastomer. This indicates that, in the elastomer matrix, some polymeric segments are orderly arrayed in a micro-domain and form small crystal grains below 40 °C. Moreover, these grains are very sensitive to temperatures lying between 25 and 40 °C, and gradually decrease in size with increasing temperature. When heated to 40 °C, the bright patches disappear completely. This indicates that elastomer S0 prepared at 20 °C is a semi-crystalline morphology elastomer below 40 °C, and an amorphous morphology elastomer above 40 °C.

**Figure 3:**
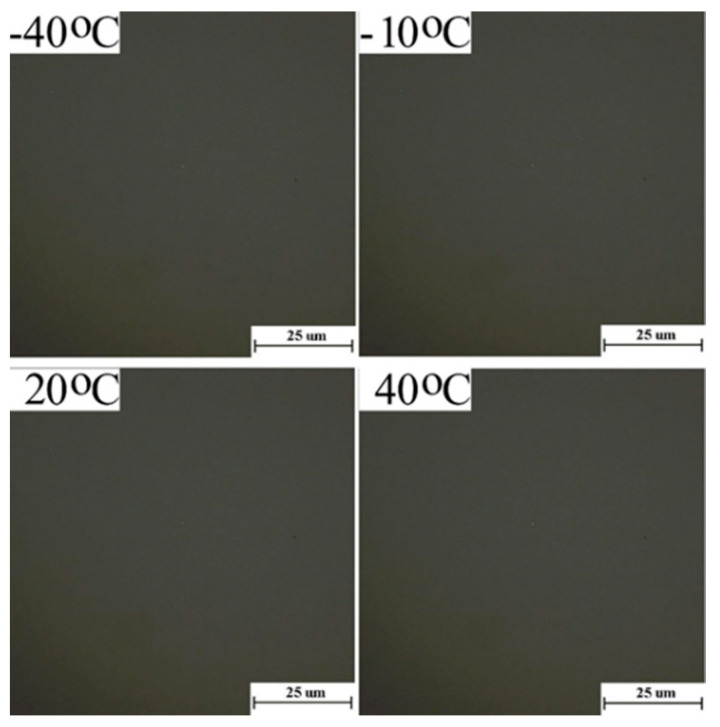
POM snapshots of elastomer S1 at different temperatures.

In contrast to the trend observed for elastomer S0, no bright patches are observed for elastomer S1 at temperatures ranging from −40 and 40 °C (see Figure 3). This indicates that elastomer S1 is characterized by an amorphous aggregation state for the temperatures considered. Taken together with the DSC curves of the elastomers (see Figure 1), these results indicate that the endothermic peak of S0 for temperatures ranging from 25 to 38 °C results from the melting of crystal grains. Moreover, semi-crystalline elastomer S0 transforms into amorphous elastomer S1 after heating, and simply reducing the temperature yields no recovery of the semi-crystalline structure.

### 3.3. Wide-Angle X-ray Diffraction

**Figure 4:**
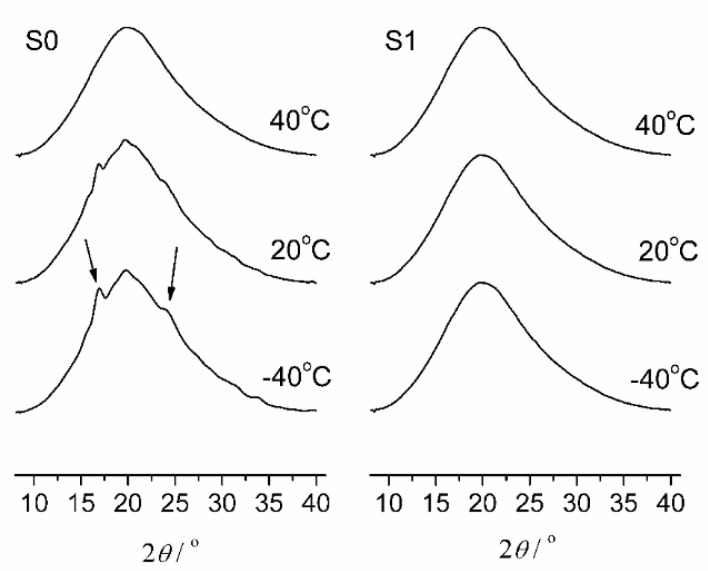
Wide-angle X-ray diffraction (WAXD) curves of elastomer S0 and S1 at different temperatures.

The crystal grain composition of elastomer S0 is determined via wide-angle X-ray diffraction (WAXD), where WAXD profiles are obtained at different temperatures (see, Figure 4 (Left)). For elastomer S0 at temperatures ranging from −40 to 20 °C, sharp diffraction peaks emerge at 16.8° and 24.1°, corresponding to the characteristic diffraction peaks of PBAMO crystallization [22]. Because BAMO random copolymerization with other monomers merely restrains (rather than eliminates) the crystallization of BAMO homo-polymerization segments [10,23,24], in combination with P(BAMO-r-THF) polymer chain structure (see Scheme 1), the small crystal grains within elastomer S0 are generated by the crystallization of BAMO micro-blocks embedded in the P(BAMO-r-THF) prepolymer. These crystalline structures persisted during preparation and preservation of the elastomer at 20 °C. In addition, the characteristic diffraction peaks of BAMO micro-block crystallization disappear completely as the temperature rises to 40 °C. This fully demonstrates the thermal effect of elastomer S0 at temperatures ranging from 25 °C to 38 °C and the POM bright patches of this elastomer below 40 °C stem from BAMO micro-block crystallization.

Figure 4 (Right) shows the WAXD profiles of elastomer S1, where a smooth halo peak occurs over the entire temperature scope. Due to the crystallization temperature reversibility of BAMO micro-blocks in the P(BAMO-r-THF) prepolymer [19], it can be concluded that the crosslinks within elastomer S1 exhibit decreased polymeric segment mobility and have kinetically restricted the rearrangement of polymerization segments. Semi-crystalline elastomer S0 becomes amorphous elastomer S1 after undergoing a heating-cooling cycle, and further spontaneous regeneration of the BAMO micro-block crystal grains, by just reducing the temperature, is prevented. The crystallinity of the BAMO micro-block within P(BAMO-r-THF) crosslinked elastomers is temperature irreversible, and hence, elastomer S1 is amorphous under static conditions.

### 3.4. Mechanical Properties

The mechanical properties obtained at different temperatures of elastomer S0 and S1 are listed in Table 2. At 20 °C, comparable break strain (*ε*_b_) values of 315 ± 22% (S0) and 294 ± 6% (S1) are obtained. However, the break stress (*σ*_b_) of S0 (0.46 ± 0.01 MPa) is obviously higher than that of S1 (0.32 ± 0.02 MPa). At −40 °C, the *ε*_b_ values increase to 1085 ± 21% for elastomer S0 and 1181 ± 25% for elastomer S1, and the corresponding *σ*_b_ values increase to 8.90 ± 0.72 MPa and 10.23 ± 0.44 MPa, respectively. In contrast to the mechanical-property at 20 °C, both elastomers simultaneously exhibit extremely high break strains and stresses at −40 °C.

**Figure 5:**
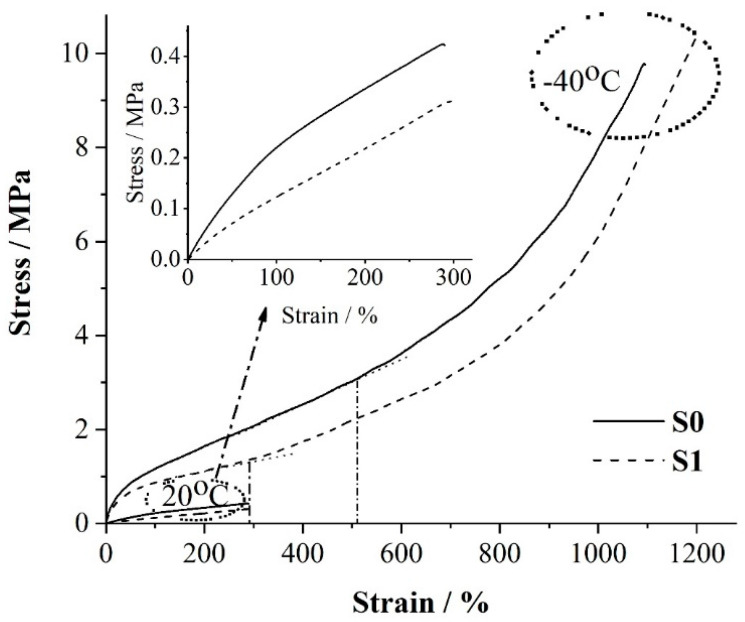
Stress–strain curves of elastomers.

The typical stress–strain curves of elastomers S0 and S1 are shown in Figure 5. At 20 °C, the stretching modulus of each elastomer decreases gradually with increasing strain, consistent with the state equation of elastomers [25]. Within elastomer S0, many BAMO micro-block crystal grains have formed physical crosslinks (see Figure 2 and Figure 4), increase the apparent strand density and reduce the apparent molecular weight of the strands between the crosslinks. The elastomer stress and modulus are proportional to the density of the cross-linked elastomer strands [26]. Therefore, in Figure 5, the stress–strain curve of elastomer S0 is considerably higher than that of elastomer S1, and the break stress of S0 higher than that of S1. Additionally, it is noteworthy that, quite different from the stress–strain trend at 20 °C, at −40 °C the stress–strain curves of elastomer S0 and S1 simultaneously give upturns of stress at strain of about 520% for elastomer S0, and at strain of about 290% for elastomer S1. And, their stress–strain behaviors are very similar.

### 3.5. Aggregation Evolution

**Figure 6:**
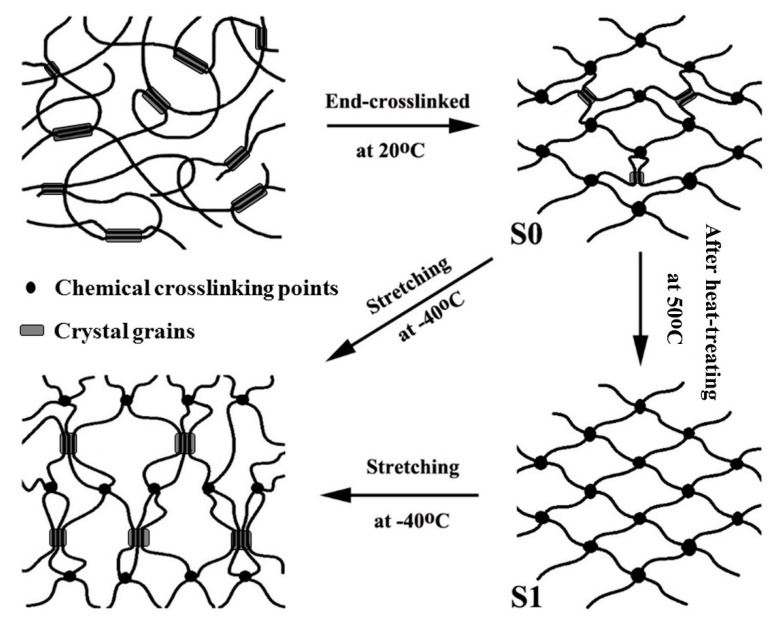
Schematic of the aggregation evolution for P(BAMO-r-THF) elastomer.

At present, a universal in-situ tensile thermal platform for synchrotron radiation X-ray diffraction is unsuitable for measuring the low-temperature mechanical properties of polymeric elastomers. To further elaborate the mechanical response mechanism of elastomer S0 and S1 at −40 °C, referring to Reference 18 as well as the POM and XRD results. Figure 6 shows the aggregation evolution process of the elastomers under different conditions.

Due to the crystallinity of BAMO micro-blocks within prepolymer P(BAMO-r-THF) at room temperature [19], elastomer S0 gives a semi-crystallization aggregation as it was prepared at 20 °C. Upon stretching at −40 °C, the strands of this elastomer slide along the stretching direction and become oriented, resulting in enhanced strand segment mobility and decreased configuration entropy [27]. The low temperature contributes to further crystallization of the other BAMO micro-blocks. The consequent strain-induced crystallization of these micro-blocks increases the physical crosslinking point density among the polymeric strands. Consequently, the modulus increases with increasing strain and an upturn of stress emerges at a strain of about 520% (see Figure 5).

After undergoing a heat-treatment at 50 °C, semi-crystalline elastomer S0 transforms into amorphous elastomer S1. The aggregation morphology of S1 is quite different from that of S0 under static conditions. However, the similar stress–strain behavior shown in Figure 5 implies that, upon stretching at −40 °C, the BAMO micro-blocks within amorphous elastomer S1 also undergo sliding and become oriented. This leads to the formation of BAMO micro-block crystal grains, with increased physical crosslinking points among the polymeric chains. Consequently, as shown in the figure, an upturn of stress at a strain of about 290% leads to an increase in the break stress. Meanwhile, strain-induced crystallization helps to elongate the segments in amorphous domains and [28], hence, the elastomers S0 and S1 simultaneously exhibit very high break strains at −40 °C. The BAMO micro-block strain-induced crystallinity yields P(BAMO-r-THF) elastomers that exhibit excellent break strain and break stress at low temperature.

## 4. Conclusions

A P(BAMO-r-THF) end cross-linked elastomer (S0) prepared at 20 °C exhibits a semi-crystallization morphology below 40 °C. After the thermal cycle, the resulting elastomer (S1) becomes completely amorphous. The crystallinity of the BAMO micro-blocks comprising the P(BAMO-r-THF) elastomer is temperature irreversible under static conditions.

At 20 °C, the BAMO micro-block crystal grains within elastomer S0 are characterized by increased crosslink density among the P(BAMO-r-THF) chains, and the modulus and break stress of this elastomer are higher than those of elastomer S1. At −40 °C, due to the dual-effect of strain orientation and low temperature, BAMO micro-blocks in both elastomers undergo strain-induced crystallization, leading to a significant improvement in the mechanical properties. These findings will contribute to extending the applications of energetic P(BAMO-r-THF) binders and stimulating the design and synthesis of new energetic binders with excellent mechanical properties.

## Figures and Tables

**Table 1:** Composition (mass ratio) of P(BAMO-r-THF) elastomer.

P(BAMO-r-THF)	N100	Dibutyltin Dilaurate
100	6.86	0.13

**Table 2:** Mechanical properties of elastomers at different temperatures.

Temperature	Sample	Mechanical Properties	1	2	3	4	Averages
20 °C	S0	*ε*_b_/%	342	318	290	310	315 ± 22
		*σ*_b_/MPa	0.47	0.45	0.44	0.46	0.46 ± 0.01
	S1	*ε*_b_/%	298	291	286	300	294 ± 6
		*σ*_b_/MPa	0.33	0.30	0.31	0.33	0.32 ± 0.02
−40 °C	S0	*ε*_b_/%	1090	1054	1096	1100	1085 ± 21
		*σ*_b_/MPa	8.85	8.03	9.78	8.94	8.90 ± 0.72
	S1	*ε*_b_/%	1179	1204	1192	1147	1181 ± 25
		*σ*_b_/MPa	9.98	10.26	10.84	9.84	10.23 ± 0.44
